# The effects of pomegranate extract on anaerobic exercise performance & cardiovascular responses

**DOI:** 10.1186/1550-2783-12-S1-P56

**Published:** 2015-09-21

**Authors:** Erica J Roelofs, Katie R Hirsch, Eric T Trexler, Meredith G Mock, Abbie E Smith-Ryan

**Affiliations:** 1 Applied Physiology Laboratory, University of North Carolina, Chapel Hill, NC, USA

## Background

During exercise, there is an increased demand for oxygen. Increasing blood flow may provide an ergogenic effect. Dietary nitrate supplementation, such as pomegranate extract (PE), has been linked to reduced vascular resistance, enhanced vasodilation, and increased blood flow to possibly improve exercise efficiency. The purpose of this study was to evaluate the effects of acute PE supplementation on anaerobic exercise, flow mediated dilation (FMD), oxygen saturation (SP0_2_), heart rate (HR), and blood pressure (BP).

## Methods

Nineteen recreationally active individuals (mean ± SD; Age: 22.1 ± 1.9 yrs; Height: 170.4 ± 12.4 cm; Weight: 68.7 ± 15.9 kg) participated in this crossover design study. In a double-blind fashion, participants were randomized to either 1000 mg of PE (True Pomegranate Extract, Stiebs Nature Elevated, Madera, CA) or placebo (PL; 95% maltodextrin, 5% purple carrot and hibiscus for color), ingested in capsule form 30 min prior to a repeated sprint ability (RSA) test. Peak and average power were identified from the RSA on a friction-loaded cycle ergometer (Monark 894E, Stockholm, Sweden), which consisted of ten six-second maximal sprints with a load of 65 g/kg of body weight with 30 seconds of passive recovery. Brachial artery FMD was assessed by ultrasound (GE logiq-e B-mode, GE Healthcare, WI) with vascular, pulse wave, and color flow settings to determine blood flow and vessel diameter. FMD, HR, SPO_2_, and BP were assessed at baseline, 30 min post ingestion (30minPI), immediately post exercise (IPost), and 30 min post exercise (30minPostEx). After a seven-day washout period, participants completed the RSA test with the opposite treatment. Separate two-way mixed factorial ANOVAs (treatment × time) were used to assess peak power, average power, FMD, BP, HR, and SPO_2_, with Bonferroni post hoc comparisons. Change scores from PE to PL were calculated and 95% confidence intervals (CI) were placed around the mean change score.

## Results

Peak power was significantly higher for sprint number 5 when supplementing with PE versus PL (mean difference [MD]= 31.81 Watts; p = 0.046) and sprint 7 trended towards significance (MD = 35.34 Watts; p = 0.063). When 95% CI were employed for peak power, sprints 5 and 7 were significantly higher when PE was consumed. Confidence intervals demonstrated average power was significantly higher for sprint 5 wth PE. Vessel diameter was significantly greater at 30minPostEx and blood flow was significantly higher IPost when PE was consumed. There were no significant differences in SP0_2_, HR, or BP.

## Conclusions

Acute supplementation of PE resulted in enhanced vessel diameter, blood flow, and repeated sprint ability halfway through the test. Results suggest the possibility of enhanced exercise performance due to increased delivery of oxygen and substrates to working skeletal muscle with the use of PE.

## Practical Applications

The acute timing and capsule form of PE may be preferable to the athletic population due to ergogenic effects, taste, and convenience. Combining PE with other ergogenic aids may be advantageous as a pre-workout supplement to further augment performance.

